# Elevated serum BMP1 in pubertal female rats and girls with central precocious puberty: a potential biomarker for longitudinal growth

**DOI:** 10.1530/EC-25-0506

**Published:** 2025-11-28

**Authors:** Yanfei Chen, Mei Li, Wei Qin, Dan Zeng, Ruiqi Wang, Jingzi Zhong, Shuting Chen, Tao Xie, Dan Lan

**Affiliations:** Department of Pediatrics, The First Affiliated Hospital of Guangxi Medical University, Nanning, China

**Keywords:** BMP1, central precocious puberty, puberty, growth, biomarker, diagnosis

## Abstract

**Objectives:**

To elucidate the expression dynamics of serum BMP1 during pubertal development and in central precocious puberty (CPP), and to evaluate its correlation with puberty-related clinical indicators and diagnostic potential for CPP.

**Animals and patients:**

Female SD rats at different developmental stages and rats with CPP models. Seventy-five girls with CPP and 55 age-matched prepubertal control girls.

**Measurements:**

Serum BMP1 levels were determined by ELISA. Spearman correlation and multivariate linear regression analyses were used to evaluate associations between BMP1 and puberty-related parameters. Receiver operating characteristic curves were plotted to evaluate the diagnostic efficacy of BMP1 for CPP.

**Results:**

Serum BMP1 levels in normal female SD rats peaked during the neonatal period and early adolescence then decreased significantly upon sexual maturity and adulthood. Markedly elevated serum BMP1 levels were observed in both CPP model rats and Tanner stage 2 girls with CPP compared to age-matched controls (*P* < 0.01). Serum BMP1 correlated positively with E2 and negatively with bone age and BA/CA ratio (*P* < 0.05). Multivariate analysis identified no independent determinants of serum BMP1 (all *P* > 0.05). For CPP diagnosis, BMP1 alone achieved an AUC of 0.692 with 76.0% sensitivity and 61.82% specificity, while the BMP1/IGF-1 combination showed superior performance (AUC = 0.832, sensitivity 84.78%, specificity 72.73%).

**Conclusions:**

Serum BMP1 demonstrates potential as a biomarker for pubertal growth but lacks sufficient diagnostic accuracy for CPP when used alone. Its combination with IGF-1 significantly improves diagnostic performance.

## Introduction

Puberty is a delicate phase orchestrated by an intricate interplay of both internal and external factors. Central precocious puberty (CPP), a prevalent endocrine condition in children, stems from the untimely activation of the hypothalamic–pituitary–gonadal (HPG) axis (1). Recent research underscores a rising trend in CPP cases (2). The progression of puberty in CPP mirrors that of typical puberty, albeit with potentially adverse effects such as stunted growth, obesity, and psychological distress (1, 2, 3, 4). Despite advancements over the decades, the intricate nuances of CPP’s causes, diagnosis, and management continue to pose significant challenges (4).

We recently conducted a proteomic analysis of serum samples from girls with CPP, which further elucidated the underlying pathophysiological mechanisms and identified potential diagnostic biomarkers with this condition (5). The findings identified 134 differentially expressed proteins (DEPs) in the serum, with 71 proteins exhibiting up-regulation and 63 showing down-regulation (5). Among these, serum bone morphogenetic protein 1 (BMP1) was notably up-regulated in girls with CPP. In the protein–protein interaction network analysis of the DEPs, BMP1 stood out with the highest fold change (FC = 2.2) among the top 25 node proteins (5). Gene ontology functional enrichment analysis indicated that BMP1 is associated with skeletal system development, extracellular region, extracellular space, and growth factor activity, which are key aspects linked to pubertal growth and development.

BMP1 is a member of the astacin family, secreted as a zinc-dependent metalloproteinase with a highly conserved structure across species (6, 7, 8). It comprises an NH2-terminal activation region, an astacin-like protease domain, several epidermal growth factor-like motifs, and a CUB protein–protein interaction domain (6). BMP1 primarily fulfills two functions: i) it coordinates extracellular matrix deposition, which includes the maturation of various procollagens and bone mineralization; and ii) it acts as an activator of multiple signaling pathways, such as the transforming growth factor β (TGF-β) and BMP signaling pathways (6). It is well established that the development of the gonads and the growth spurt are the two most prominent features of puberty (9). All aforementioned functions of BMP1 are implicated in the regulation of longitudinal bone growth (10). Furthermore, the TGF-β and BMP signaling pathways play crucial roles in the HPG axis, including the regulation of gonadotropin-releasing hormone, gonadotropin secretion, and follicular development (11, 12, 13, 14). Studies also suggest that BMP1 is involved in the development and maturation of various mammalian follicles (15, 16). Considering these findings and proteomic evidence of elevated serum BMP1 levels in girls with CPP, we hypothesize that serum BMP1 is closely related to pubertal development. However, to our knowledge, no direct studies have explored this relationship.

In this study, our objective was to further elucidate the expression of BMP1 during puberty and in CPP, preliminarily explore the potential association between serum BMP1 and puberty, and assess its feasibility as a diagnostic biomarker for CPP. We profiled serum BMP1 across developmental stages in normal female rats and evaluated BMP1 levels in a CPP rat model and in girls with CPP. We also analyzed the correlation between serum BMP1 and puberty-related clinical indicators. Finally, we assessed the diagnostic efficacy of serum BMP1 as a potential biomarker for CPP.

## Methods

### Normal female SD rats at different stages of development

To preliminarily explore the relationship between serum BMP1 and normal sexual development, we first attempted to determine the expression profile of serum BMP1 at different developmental stages. Given that the developmental process in rodents is similar to that in humans, we selected normal female Sprague–Dawley (SD) rats as the subjects of our study. We selected six developmental stages in total (*n* = 9–10 per group): neonatal period (postnatal day 4, PND4), infant period (PND14), peripubertal period (PND27), early adolescence (first day of vaginal opening), sex maturity (first regular estrous), and adulthood (PND67). It is noted that vaginal opening is an external marker for the onset of puberty in rats (17), and thus the first day of vaginal opening was defined as early adolescence. After weaning (around PND21), normal female rats were observed daily for vaginal opening to determine the exact age at which it occurred. Once vaginal opening was observed, the vaginal area was washed daily with saline to collect exfoliated cells for smear examination. This procedure was specifically employed to ascertain the precise age at which the first regular estrous cycle emerged, a milestone that marks the maturation of puberty in rats (17).

### Establishment of CPP rat model

The CPP rat model is widely utilized as an experimental framework for investigating the pathogenesis of precocious puberty. In this study, we developed a danazol-induced CPP rat model to examine alterations in serum expression associated with CPP. The methodology was as follows: 5-days-old female SD rats (*n* = 16) were randomly chosen for a subcutaneous injection of 300 μg of danazol (Solarbio, China). The control group (*n* = 16) received the same volume of normal saline. Blood samples were collected from the orbital sinus of the CPP model group during their first estrous period. Concurrently, blood samples were randomly collected from the same-age control group (*n* = 8), designated as control group 1. The remaining control group rats, designated as control group 2 (*n* = 8), were monitored for the timing of vaginal opening and the onset of the first estrous period. The advancement of vaginal opening and the first estrous cycle, along with elevated sex hormone levels in the CPP model group compared to the controls, confirmed the successful establishment of the CPP rat model.

### Measurement of serum BMP1 and sex hormones in SD rats

All the female SD rats were purchased from Guangxi Medical University (Nanning, China) and were housed under standard conditions: a 12 h light:12 h darkness cycle and a temperature of 22°C. Orbital blood samples were collected from female rats of different ages and CPP model rats, and the serum was separated by centrifugation (1,811 ***g***, 15 min) and stored at −80°C. Serum levels of BMP1, luteinizing hormone (LH), and estradiol (E2) in SD rats were measured by commercial enzyme-linked immunosorbent assay (ELISA) kits. Serum BMP1 concentrations were determined using a Rat BMP1 ELISA Kit (Jianglai, Shanghai). All samples were analyzed in duplicate, with a minimum detectable level of 15.62 pg/mL. The assay exhibited intra- and inter-assay coefficients of variation (CV) below 10%, and a mean recovery rate of 98%. For serum E2 and LH quantification, corresponding rat ELISA kits (Fine Test, Wuhan) were employed. The E2 assay had a detection limit of 12.5 pg/mL, with intra- and inter-assay CVs of <5.91% and <5.85%, respectively. The LH assay demonstrated a detection limit of 0.313 mIU/mL, with intra- and inter-assay CVs of <6.43% and <5.22%, respectively.

### Patients and laboratory measurement assessments

This study was conducted from September 2019 to November 2024 in the First Affiliated Hospital of Guangxi Medical University (Nanning, China), and 75 girls with CPP and 55 prepubertal healthy girls of similar age, defined as normal controls during routine checkups, were recruited. Among the CPP group, 50, 15, and 10 girls were classified as Tanner stages 2, 3, and 4, respectively, based on their breast development. All children with CPP met the diagnostic criteria of breast development before the age of 7.5 years, accompanied by accelerated growth (≥6 cm/year), progressive increase in bone age, and the peak level of LH greater than 5 mIU/mL (1). Girls in the control group showed no signs of puberty, including breast development, growth spurt, development of pubic and axillary hair, or the onset of menarche. All research participants did not include girls with chronic illnesses, thyroid dysfunctions, central organic brain disorders, congenital adrenal hyperplasia, and other endocrine irregularities.

A qualified pediatric endocrinologist booked the anthropometric parameters of the participants, including their height, weight, and Tanner stage of breast development. Moreover, girls with CPP underwent bone age assessment (BA) and ultrasound scans to evaluate uterine and ovarian conditions. Body mass index (BMI) was defined as weight in kilograms divided by height in meters squared (kg/m^2^).

Blood samples were collected for the CPP group before the administration of a dose of triptorelin (Ferring, Germany) at 2.5 μg/kg (maximum dose, 100 μg). Subsequently, blood sampling was performed at 30, 60, 90, and 120 min after hormone administration. Human serum BMP1 levels were assessed using a human BMP1-specific ELISA kit (Fine Test, Wuhan). Duplicate measurements were performed, yielding a detection limit of 78.125 pg/mL, intra- and inter-assay CVs of <5.42% and <5.3%, and a recovery rate of 95%. No significant cross-reactivity was observed, which validated the assay’s specificity. Serum concentrations of LH, E2, follicle-stimulating hormone (FSH), and insulin-like growth factor 1 (IGF-1) were measured using chemiluminescence immunometric assays (Mindray, CL-2000i, China).

### Statistical analysis

We employed IBM SPSS Statistics version 25.0 to analyze our dataset. Initially, we checked the normality of the data using the Shapiro–Wilk test. For variables adhering to a normal distribution, we conducted an ANOVA for comparisons. Conversely, for those variables deviating from normal distribution, we applied the Mann–Whitney U test and Kruskal–Wallis H test. Spearman’s rank correlation analysis was employed to evaluate the univariate associations between serum BMP1 levels and other clinical parameters, including hormonal profiles, bone age, and anthropometric measures. Variables that demonstrated significance in univariate analysis, or were of established clinical relevance, were subsequently entered into a multivariate linear regression model to identify independent determinants of serum BMP1, with multicollinearity assessed by variance inflation factors (VIF). In addition, receiver operating characteristic (ROC) curve analysis was performed to evaluate the diagnostic potential of serum BMP1 for CPP, from which the optimal cutoff value and its corresponding sensitivity and specificity were derived. To further enhance the diagnostic efficacy, a combined prediction model incorporating both BMP1 and IGF-1 was developed and subjected to ROC analysis. A two-tailed *P*-value <0.05 was considered statistically significant.

## Results

### Dynamic changes in serum BMP1, LH, and E2 levels in female SD rats at various developmental stages

**Figure 1:**
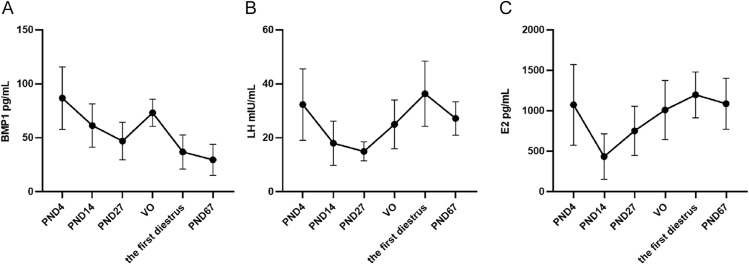
Serum BMP1, LH, and E2 levels in normal female SD rats at different developmental stages. PND, postnatal day; VO, vaginal opening.

From birth through adulthood, we observed two peaks in serum BMP1 levels: one in the neonatal period (PND4) and another at the onset of puberty (marked by vaginal opening). Expression of BMP1 then significantly declined during sexual maturation and remained lower in adulthood. Similarly, serum LH and E2 levels also peaked twice – first in the neonatal period and again at the onset of sexual maturity (first diestrus) – but unlike BMP1, they maintained higher levels of expression in adulthood, as shown in Fig. 1.

### Serum BMP1 levels in female rats with CPP

**Figure 2:**
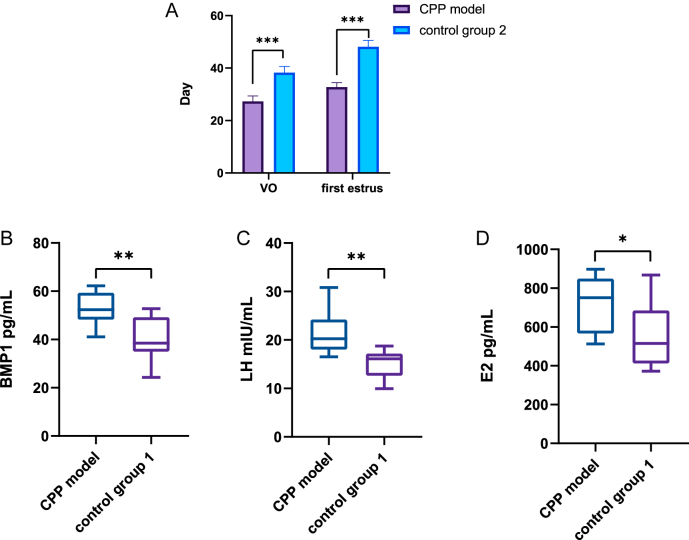
VO, first estrous cycle, and serum indices in CPP model versus controls. (A) CPP model: VO 27.3 ± 2.1 d, first estrous 32.8 ± 1.6 d; both significantly earlier than control 2 (VO 38.3 ± 2.4 d; cycle 48.1 ± 2.5 d). (B, C, D) Serum BMP1, LH, and E2 concentrations were significantly elevated in the CPP model compared with control group 1. Statistical significance: *P* < 0.05, *P < 0.01, **P < 0.001; ns, *P* > 0.05.

The vaginal opening time (27.3 ± 2.05 days) and first estrous cycle (32.80 ± 1.61 days) in the model group were significantly earlier than those in control group 2 (*P* < 0.001). Compared to control group 1, the CPP rat model exhibited significantly higher levels of LH and E2 (*P* < 0.05), confirming the successful establishment of the precocious puberty model. In addition, BMP1 expression was significantly upregulated in the precocious puberty rat model, as shown in Fig. 2.

### Anthropometric measurements and serum BMP1 levels in girls with CPP

**Figure 3:**
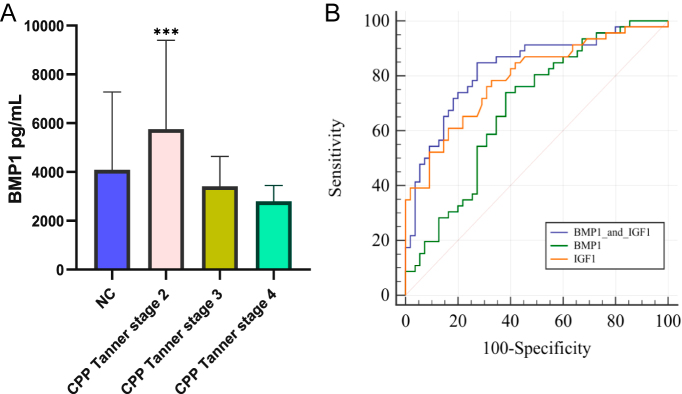
Diagnostic value of serum BMP1 and IGF-1 for CPP. (A) Serum BMP1 levels in girls with CPP at Tanner stage 2 compared to prepubertal controls and Tanner stages 3–4 groups (***P < 0.001). (B) ROC curves analyzing the diagnostic performance of BMP1 alone, IGF-1 alone, and their combination for identifying CPP. BMP1 achieved an area under the curve (AUC) of 0.692 (95% CI: 0.594–0.778, *P* < 0.01) with 76.0% sensitivity and 61.82% specificity at the optimal cutoff of 3,165.66 pg/mL; IGF-1 yielded an AUC of 0.791 (95% CI: 0.698–0.865, *P* < 0.01) with 78.26% sensitivity and 67.27% specificity at 218.5 ng/mL; the combined model showed improved discrimination with an AUC of 0.832 (95% CI: 0.744–0.899, *P* < 0.01), 84.78% sensitivity, and 72.73% specificity.

There was no significant age difference between girls with CPP and the control group (*P* > 0.05). However, the CPP group showed significantly higher levels of height, weight, basal LH, basal FSH, E2, and IGF-1 compared to the prepubertal controls (*P* < 0.01), indicating advanced physical development and elevated sex hormone levels in CPP girls. Notably, serum BMP1 was significantly higher in CPP girls at Tanner stage 2 (early puberty) than in both prepubertal controls and those at Tanner stages 3–4 (mid-to-late puberty) (*P* < 0.001, Table 1 and Fig. 3).

**Table 1:** Anthropometric and laboratory parameters of the groups.

Items	NC (*n* = 55)	CPP (*n* = 75)			*P* value
		Tanner stage 2	Tanner stage 3	Tanner stage 4	
		(*n* = 50)	(*n* = 15)	(*n* = 10)	
Age (years)	7.47 ± 0.90	7.57 ± 0.78	7.72 ± 1.04	8.25 ± 1.05	0.082
Bone age (years)	—	8.68 ± 1.37	9.3 ± 1.16	11 ± 1.41	<0.001
Height (cm)	122.23 ± 5.84	129.45 ± 6.78	136.04 ± 10.54	140.12 ± 9.26	<0.001
Weight (kg)	22.19 ± 3.18	27.19 ± 4.94	33.8 ± 8.18	37.62 ± 13.95	<0.001
BMI (kg/m^2^)	14.96 ± 1.55	16.11 ± 1.75	17.99 ± 2.17	18.96 ± 2.89	<0.001
Uterus volume (mL)	—	2.85 (1.74, 3.73)	4.69 (2.26, 7.95)	4.96 (3.89, 7.04)	<0.001
Ovarian volume (mL)	—	2.26 (1.43, 3.28)	2.30 (1.43, 4.48)	3.09 (2.39, 4.13)	0.178
LH (mIU/mL)	0.11 (0.07, 0.15)	0.65 (0.28, 1.26)	2.02 (0.75, 2.43)	3.77 (2.41, 6.22)	<0.001
FSH (mIU/mL)	1.79 (1.42, 2.29)	3.05 (2, 5.31)	3.99 (2.7, 5.71)	5.46 (2.83, 6.47)	<0.001
E2 (pmol/L)	15.51 (6.43, 21.79)	27.65 (19.19, 35.04)	29.46 (20.59, 54.38)	16.71 (5.27, 58.46)	<0.001
IGF-1 (nmol/L)	184.3 (147, 245)	301.00 (226.45, 403.9)	346.15 (257.77, 392.97)	340.3 (269.02, 404.50)	<0.001
**BMP1 (pg/mL)**	**2,812.38 (1,926.46, 5,238.82)**	**4,228.75 (3,209.71, 8,588.38)**	**3,207.98 (2,911.64, 3,667.02)**	**2,785.3 (2,440.47, 3,283.22)**	**0.006**

NC, normal control; CPP, central precocious puberty; CA, chronological age; BA, bone age. Data are presented as mean ± SD for normally distributed variables and as median (interquartile range) for non-normally distributed variables.

### Correlation and multivariate analyses

Spearman correlation analysis revealed a positive correlation of serum BMP1 level with E2 level, but a negative correlation with bone age and the ratio of bone age/chronological age (*P* < 0.05). Statistically significant correlations were not detected for serum BMP1 levels with chronological age, height, weight, BMI, uterine volume, ovarian volume, basal LH, basal FSH, peak FSH, peak LH, and IGF-1 (*P* > 0.05), as displayed in Table 2.

**Table 2:** The correlation analysis between the serum BMP1 levels and clinical parameters.

Parameters	*n*	*R* value	*P* value
Age (years)	130	−0.027	0.764
**Bone age (years)**	**75**	**−0.380**	0.001*
**BA/CA**	**75**	**−0.336**	0.003*
Height (cm)	130	0.027	0.764
Weight (kg)	130	0.036	0.688
BMI (kg/m^2^)	130	0.028	0.092
Uterus volume (mL)	74	−0.223	0.057
Ovarian volume (mL)	74	−0.185	0.115
**E2 (pmol/L)**	**130**	**0.231**	0.008*
LH (mIU/mL)	130	0.104	0.239
FSH (mIU/mL)	127	0.131	0.142
P-LH (mIU/mL)	75	−0.215	0.064
P-FSH (mIU/mL)	75	0.078	0.507
IGF-1 (nmol/L)	121	0.061	0.510

CA, chronological age; BA, bone age; P-, peak. Correlation coefficients were computed using Spearman’s correlation. *P* < 0.05 was considered statistically significant.

* Indicates statistical significance.

In the multivariate model, VIFs ranged from 1.0 to 4.0, indicating negligible collinearity. Nevertheless, none of the included variables (age, BMI, E2, IGF-1, bone age, uterine/ovarian volume, basal or peak gonadotropins) exhibited a statistically significant regression coefficient for serum BMP1 (all *P* > 0.05). Consequently, fluctuations in serum BMP1 appear to result from the weak, cumulative effects of multiple factors rather than from any single measurable determinant, as shown in Table 3.

**Table 3:** Determinants of serum BMP1 levels from a multivariate linear regression analysis.

Variable	*β* (95% CI)	*P* value	VIF
Age (years)	0.003 (−1.377, 1.383)	0.997	1.971
Bone age (years)	0.191 (−0.868, 1.250)	0.719	3.689
BMI (kg/m^2^)	−0.201 (−0.694, 0.292)	0.417	1.794
Uterus volume (mL)	−0.243 (−2.062, 0.577)	0.555	3.736
Ovarian volume (mL)	0.308 (−0.509, 1.125)	0.453	2.095
LH (mIU/mL)	−0.133 (−0.949, 0.682)	0.744	3.959
FSH (mIU/mL)	−0.083 (−0.698, 0.533)	0.789	2.436
E2 (pmol/L)	−0.025 (−0.092, 0.041)	0.450	1.404
P-LH (mIU/mL)	−0.019 (−0.092, 0.055)	0.609	2.456
P-FSH (mIU/mL)	0.042 (−0.099.0.183)	0.557	1.461
IGF-1 (nmol/L)	−0.008 (−0.017, 0.001)	0.090	1.212

Note. Dependent variable: BMP1; intercept = 10.249 (SE = 5.736, *P* = 0.08).

### ROC analysis

ROC curve analysis was performed to evaluate the diagnostic value of serum BMP1 and IGF-1 in distinguishing girls with CPP from normal controls. Serum BMP1 demonstrated an area under the curve (AUC) of 0.692 (95% CI: 0.594–0.778, *P* < 0.01), with an optimal cutoff value of 3,165.66 pg/mL corresponding to a sensitivity of 76.0% and a specificity of 61.82%. IGF-1 showed an AUC of 0.791 (95% CI: 0.698–0.865, *P* < 0.01), with an optimal cutoff value of 218.5 ng/mL providing a sensitivity of 78.26% and a specificity of 67.27%. Furthermore, the combination of BMP1 and IGF-1 exhibited enhanced diagnostic performance, achieving an AUC of 0.832 (95% CI: 0.744–0.899, *P* < 0.01) with a sensitivity of 84.78% and a specificity of 72.73%, as shown in Fig. 3.

## Discussion

As a metalloproteinase, BMP1 is pivotal in morphogenesis, aiding in the maturation of various extracellular matrix proteins and activating specific growth factors within the TGF-β superfamily (6). Previous proteomic analysis revealed a marked increase in BMP1 levels in cases of CPP, indicating that this proteinase might have a significant involvement in puberty and CPP (5).

### Serum BMP1 could serve as a marker for longitudinal growth

In this study, we first assessed the pattern of BMP1 changes in normal SD female rats. The results showed that serum BMP1 expression increased during early puberty, further proving that serum BMP1 is associated with puberty. We also observed that the changes in serum BMP1 expression were similar to those of serum LH and E2, with peaks appearing in the neonatal and pubertal periods. However, during the pubertal stage, serum BMP1 peaked earlier than sex hormones; in sexual maturity and adulthood, serum BMP1 expression significantly decreased, while sex hormones maintained a certain level of expression. These results suggest that sex hormones may be associated with the expression of serum BMP1, but this connection is not continuous.

We further observed that the changes in serum BMP1 levels closely match the pattern of longitudinal growth, which is mainly driven by bone growth. This growth pattern is fastest in infancy, slows down gradually in childhood, speeds up again during puberty, and stops in adulthood (18). Moreover, longitudinal growth during adolescence progresses through three stages: an initial acceleration (take-off), a peak, and then a deceleration (18, 19). Similarly, serum BMP1 levels were highest during the neonatal period, slightly decreased during childhood, increased again in early adolescence, then declined in late adolescence (after sexual maturation), and reached the lowest levels in adulthood.

Previous studies have highlighted the critical importance of BMP1 in bone growth and development. The primary driver of longitudinal bone growth is the elongation of the epiphyseal growth plate, a process that is intricately regulated by chondrocytes through a series of biological events, including proliferation, hypertrophy, cartilage matrix synthesis, and mineralization (20). BMP1, expressed in growth plate tissues (8), is crucial for matrix formation in the growth plate (6). It promotes the maturation of type II procollagen by cleaving its C-propeptide, a key structural component of growth plate cartilage (6). In addition, microarray gene expression analysis has shown that BMP1 expression increases during the late stages of growth plate damage repair, which suggests BMP1 is also involved in the repair and maintenance of the growth plate (21). Beyond its role in collagen maturation, BMP1 acts as an activator of the TGF-β and BMP pathways, both of which are crucial for bone development (6, 10). BMP1 significantly enhances the alkaline phosphatase activity of bone marrow stromal cells (BMSCs), increases intracellular Ca^2+^ concentration, promotes collagen expression, and fosters osteogenic differentiation (22). Conversely, BMP1 deficiency has been shown to lead to osteogenesis imperfection, characterized by recurrent fractures, bone malformations, bone density abnormalities, and growth plate fracture and widening, ultimately resulting in growth retardation (23). Previous studies have demonstrated a close relationship between BMP1 and bone development. In line with these findings, our study shows that changes in serum BMP1 levels closely mirror the pattern of longitudinal growth. This suggests that monitoring serum BMP1 could offer a rapid and sensitive way to reflect changes in growth velocity. Given the importance of tracking growth velocity in child development – it allows for early detection of growth abnormalities and potential health issues, such as malnutrition, delayed puberty, or precocious puberty (24) – our findings highlight the potential value of serum BMP1 as a biomarker. This result warrants further exploration.

Consistent with previous proteomics studies (5), this study further demonstrated that serum BMP1 expression was elevated in both CPP rat models and CPP girls, as assessed by ELISA. Mirroring the trend seen in normal female SD rats, we observed that serum BMP1 levels were significantly increased in girls with CPP during early adolescence (Tanner stage 2) but decreased during mid-to-late adolescence (Tanner stages 3 and 4). This pattern suggests that serum BMP1 expression in CPP girls may also be closely linked to longitudinal growth. Therefore, the increased serum BMP1 levels in CPP girls may be attributed to the onset of puberty, which leads to a growth rate faster than that of their age-matched peers.

### Correlation analysis

This study examined the correlation between serum BMP1 and clinical indicators of puberty to further investigate the potential role of BMP1 during puberty. The results revealed a positive correlation between serum BMP1 and E2. E2 is the primary hormone responsible for the growth spurt during puberty. Previous studies have indicated that the expression of BMP1 may be regulated by E2. In an ovariectomized mouse model of osteoporosis, treatment with E2 significantly upregulated the expression of BMP1 in the metaphyseal tissues (25). In light of the positive correlation between BMP1 and E2 levels observed in this study, BMP1 may play a significant role in bone growth processes mediated by E2.

We found that serum BMP1 levels in girls with CPP are negatively correlated with bone age, which reflects the degree of growth plate aging and is closely related to residual growth potential (26). This result indicates that serum BMP1 levels may serve as a potential indicator of growth plate aging. Under normal physiological conditions, the aging of the growth plate is accompanied by a series of cellular and tissue changes: blood vessels and osteoblasts grow and migrate inward, the number of chondrocytes gradually decreases, and the extracellular matrix (ECM) secreted by chondrocytes is eventually replaced by bone tissue (20). BMP1 is a crucial protease involved in ECM formation in the growth plate (6). Therefore, we speculate that the negative correlation between BMP1 and bone age may be due to the gradual reduction of ECM during growth plate aging, which is accompanied by decreased BMP1 expression.

This study found no significant correlation between serum BMP1 levels and puberty-related indicators such as ovarian volume, LH, and FSH. The substrates activated by BMP1, such as TGF-β and BMP signal, are closely related to the regulation of the HPG axis (11, 12, 13, 14). A number of studies have reported that BMP1 is also involved in regulating ovarian development by promoting oocyte maturation and granulosa cell proliferation (15, 16). These findings suggest that BMP1 may play a role in the central initiation of puberty and ovarian development. However, correlation analysis in this study showed little correlation between serum BMP1 changes and these processes.

### ROC analysis

The search for effective and stable indicators for the early diagnosis of CPP remains a research hotspot (27). Recently, biomarkers related to bone metabolism, such as IGF-1 and osteocalcin, have shown significant potential in the early diagnosis, subtype classification, and assessment of therapeutic efficacy in girls with CPP (28, 29, 30). Thus, we further evaluated the performance of BMP1 in the diagnosis of CPP using ROC curves. The results indicated that serum BMP1 exhibited high sensitivity but low specificity in distinguishing CPP from normal controls, suggesting limitations in its standalone application. Notably, the combination of BMP1 and IGF-1 significantly improved diagnostic performance, achieving an area under the curve of 0.832, with 84.78% sensitivity and 72.73% specificity. This finding indicates that a multi-marker strategy may provide a more reliable laboratory basis for early CPP identification.

The relatively low diagnostic specificity of serum BMP1 may be attributed to its close association with growth velocity. According to our research hypothesis, BMP1 primarily reflects the body’s longitudinal growth rate, and there exists significant heterogeneity in growth patterns among children with CPP: those with slowly progressive CPP (SP-CPP) typically maintain normal growth velocity, whereas children with rapidly progressive CPP (RP-CPP) may eventually exhibit decreased growth velocity in advanced stages due to accelerated bone age maturation (31, 32). This heterogeneity in growth patterns leads to substantial overlap in BMP1 expression levels between children with CPP and normal controls, thereby limiting its specificity when used alone for CPP diagnosis.

### Limitations and future directions

It should be specifically noted that the cross-sectional design of this study presents an important methodological limitation: due to the failure to systematically evaluate growth velocity – a key clinical parameter – we cannot accurately determine the specific discriminatory value of BMP1 for RP-CPP. Notably, accelerated growth is typically a characteristic feature of the early stages of RP-CPP, and BMP1 levels may reflect such changes in growth velocity. Therefore, BMP1 may be more suitable as a serological marker for the early identification of RP-CPP rather than for predicting general CPP. To validate this hypothesis, prospective cohort studies are strongly recommended to systematically monitor dynamic changes in serum BMP1 and growth velocity data from the prepubertal stage until final height is reached.

In addition, this study has the following limitations: the sample size was limited, requiring expansion to validate the expression patterns of BMP1 during puberty; the clinical application value of BMP1 still needs confirmation, particularly its potential as an indicator for assessing the efficacy of GnRHa treatment, previous research has shown that GnRHa therapy can reduce bone turnover markers in children with CPP by inhibiting osteoblast activity (29). Finally, our findings suggest that serum BMP1 levels may reflect growth plate aging, and whether BMP1 can help assess remaining growth potential in girls with CPP and thus provide a basis for clinical intervention warrants further investigation.

## Conclusion

This study confirms that serum BMP1 is closely associated with longitudinal growth during puberty and may serve as a potential biomarker reflecting growth velocity. Although its value as a standalone diagnostic marker for CPP is limited, its combination with IGF-1 significantly improves diagnostic accuracy. Future research should focus on two main directions: first, exploring the feasibility of BMP1 as an early identification marker for RP-CPP; second, evaluating its clinical application value in predicting remaining growth potential in children with CPP.

## Declaration of interest

The authors declare that there is no conflict of interest that could be perceived as prejudicing the impartiality of the work reported.

## Funding

The funds for this research were obtained from the Beijing Integrated Medicine Association (Grant No. ZHKY-2024-3314) and Guangxi Clinical Research Center for Pediatric Disease (No: AD22035219), the Key Laboratory of Children’s Disease Research in Guangxi's Colleges and Universities, and the Open Project of Guangxi Key Laboratory of Precision Medicine for Genetic Diseases (Maternal and Child Health Hospital of Guangxi Zhuang Autonomous Region) (Grant No. GXWCH-ZDKF-2022-18).

## Author contribution statement

Y Chen and M Li completed the experimental portion of this study and drafted the manuscript. D Lan participated in study design, directed the experimental operation, analyzed the experimental results, and modified the manuscript. W Qin, R Wang, D Zeng, J Zhong, S Chen, and T Xie performed the specimen collection. All the authors approved the final version of the manuscript.

## Statement of ethics

This study was approved by the Research Ethics Committee of the First Affiliated Hospital of Guangxi Medical University (approval No. 2023-E539-01) and the Laboratory Animal Welfare and Ethics Committee of Guangxi Medical University (approval No. 202501003). It was conducted in accordance with the Declaration of Helsinki, and informed consent was obtained from all participants’ parents.
